# Supplementary material for: Growth and metal bioconcentration by conspecific freshwater macroalgae cultured in industrial waste water
Source: PeerJ. 2014 May 22;2:e401. doi: 10.7717/peerj.401 (PMC4034596; doi:10.7717/peerj.401)
Supplement: Table S1 [file peerj-02-401-s001.docx]

**Table S1** Concentration (mg kg^-1^) of elements in the three *Oedogonium* species before (initial) and after 1-3 weeks of cultivation in Ash Dam water.

|  | KC 606974 | | | | KC 606977 | | | | KC 701473 | | | |
| --- | --- | --- | --- | --- | --- | --- | --- | --- | --- | --- | --- | --- |
|  | Initial | Week 1 | Week 2 | Week 3 | Initial | Week 1 | Week 2 | Week 3 | Initial | Week 1 | Week 2 | Week 3 |
| Al | 4.34 ± 0.38 | 28.4 ± 10.9 | 73.3 ± 25.2 | 28.1 ± 2.5 | 24.3 ± 12.8 | 32.2 ± 9.1 | 28.3 ± 5.2 | 40.9 ± 1.2 | 8.90 ± 5.67 | 2.09 ± 1.84 | 29.6 ± 9.5 | 41.0 ± 7.8 |
| As | < LOD | 20.1 ± 1.0 | 20.1 ± 1.5 | 15.6 ± 1.1 | < LOD | 17.1 ± 2.1 | 15.4 ± 2.2 | 16.7 ± 1.5 | 0.69 ± 0.19 | 21.6 ± 11.1 | 35.1 ± 1.8 | 35.8 ± 5.6 |
| B | 7.44 ± 1.69 | 138.3 ± 18.4 | 344.7 ± 51.8 | 231.2 ± 84.1 | 20.0 ± 0.9 | 92.3 ± 18.0 | 103.6 ± 27 | 192.5 ± 58.9 | 9.22 ± 1.14 | 47.5 ± 22.3 | 63.9 ± 2.7 | 84.2 ± 5.3 |
| Ba | 37.5 ± 6.0 | 185.0 ± 33.2 | 172.6 ± 38.4 | 64.3 ± 12.7 | 34.3 ± 2.9 | 55.1 ± 8.9 | 21.7 ± 3.9 | 16.0 ± 3.3 | 67.8 ± 8.4 | 125.7 ± 80.3 | 94.2 ± 59.6 | 69.4 ± 22.9 |
| Ca | 2,670 ± 514 | 5,170 ± 143 | 6,560 ± 147 | 5,003 ± 987 | 3063 ± 434 | 2463 ± 223 | 2517 ± 404 | 3,100 ± 407 | 2,847 ± 448 | 3,387 ± 508 | 3,630 ± 236 | 3,617 ± 309 |
| Cd | < LOD | 1.22 ± 0.17 | 2.38 ± 0.43 | 1.21 ± 0.17 | 0.03 ± 0.00 | 1.97 ± 0.30 | 1.69 ± 0.23 | 1.82 ± 0.25 | 0.06 ± 0.03 | 0.30 ± 0.20 | 0.71 ± 0.07 | 0.98 ± 0.28 |
| Co | 0.08 ± 0.02 | 1.42 ± 0.03 | 1.45 ± 0.13 | 0.63 ± 0.03 | 0.39 ± 0.01 | 0.51 ± 0.01 | 0.49 ± 0.05 | 0.57 ± 0.08 | 0.16 ± 0.01 | 0.74 ± 0.35 | 1.69 ± 0.28 | 1.01 ± 0.12 |
| Cr | 0.36 ± 0.08 | 0.40 ± 0.04 | 0.47 ± 0.05 | 0.39 ± 0.03 | 0.27 ± 0.02 | 0.66 ± 0.15 | 0.35 ± 0.06 | 0.34 ± 0.00 | 0.73 ± 0.07 | 0.56 ± 0.10 | 0.39 ± 0.04 | 0.51 ± 0.09 |
| Cu | 5.48 ± 1.08 | 10.24 ± 0.32 | 9.57 ± 0.40 | 6.85 ± 0.45 | 5.65 ± 0.09 | 8.76 ± 0.70 | 6.72 ± 0.08 | 5.33 ± 0.44 | 9.75 ± 1.02 | 8.57 ± 1.81 | 7.39 ± 0.47 | 10.9 ± 1.0 |
| Fe | 590.7 ± 117 | 2,417 ± 185 | 2,303 ± 95 | 1,570 ± 85 | 530.7 ± 53 | 1,180 ± 85 | 1,147 ± 27 | 1,042 ± 152 | 940.0 ± 51.9 | 1,429 ± 596 | 1,670 ± 205 | 2,150 ± 386 |
| K | 13,387 ± 2,700 | 11,637 ± 1,309 | 8,593 ± 1,751 | 11,017 ± 1,099 | 10,283 ± 773 | 9,650 ± 793 | 8,447 ± 1,454 | 6,767 ± 315 | 25,533 ± 1,433 | 19,167 ± 3,002 | 15,533 ± 2,384 | 14,193 ± 2,678 |
| Mg | 2,283 ± 416 | 2,947 ± 245 | 4,187 ± 1,282 | 2,370 ± 115 | 2,280 ± 180 | 4,043 ± 979 | 2,610 ± 315 | 4,373 ± 1,075 | 2,900 ± 67 | 2,910 ± 388 | 3,007 ± 175 | 2,930 ± 568 |
| Mn | 14.1 ± 2.8 | 68.8 ± 5.58 | 50.5 ± 9.0 | 33.5 ± 2.39 | 15.0 ± 2.21 | 25.1 ± 2.95 | 25.7 ± 0.54 | 19.9 ± 1.74 | 35.33 ± 3.35 | 64.5 ± 8.3 | 75.2 ± 6.7 | 60.9 ± 10.9 |
| Mo | 1.00 ± 0.12 | 5.89 ± 0.80 | 7.70 ± 0.57 | 5.12 ± 0.77 | 6.73 ± 0.78 | 4.28 ± 0.52 | 3.37 ± 0.58 | 2.89 ± 0.26 | 1.43 ± 0.29 | 4.47 ± 1.13 | 4.84 ± 0.32 | 4.15 ± 0.42 |
| Na | 6,430 ± 1,262 | 1,947 ± 446 | 2,413 ± 225 | 1,650 ± 191 | 672.7 ± 35.5 | 1,497 ± 181 | 1,350 ± 218 | 1,477 ± 202 | 6,657 ± 1,097 | 7,180 ± 4,910 | 1,927 ± 126 | 1,567 ± 62 |
| Ni | 0.38 ± 0.06 | 31.5 ± 3.1 | 59.6 ± 6.4 | 39.7 ± 4.2 | 0.40 ± 0.05 | 28.3 ± 0.8 | 30.6 ± 7.9 | 33.6 ± 3.8 | 0.62 ± 0.10 | 8.69 ± 5.13 | 39.5 ± 2.6 | 48.5 ± 7.9 |
| P | 2,263 ± 453 | 5,310 ± 446 | 4,300 ± 374 | 2,913 ± 224 | 1,690 ± 95 | 3,503 ± 155 | 2,543 ± 71 | 2,157 ± 308 | 4,520 ± 289 | 6,317 ± 1,418 | 4,937 ± 84 | 4,483 ± 355 |
| Pb | 0.15 ± 0.03 | 1.82 ± 0.80 | 0.74 ± 0.25 | 0.47 ± 0.14 | 0.18 ± 0.02 | 0.33 ± 0.16 | 0.48 ± 0.22 | 0.22 ± 0.05 | 0.24 ± 0.05 | 0.58 ± 0.39 | 0.49 ± 0.25 | 0.24 ± 0.02 |
| S | 670.7 ± 175.5 | 3,463 ± 77 | 4,510 ± 142 | 3,327 ± 364 | 279.3 ± 51.2 | 2,913 ± 180 | 2,813 ± 651 | 4,067 ± 662 | 1,650 ± 132 | 2,311 ± 938 | 2,840 ± 207 | 3,387 ± 472 |
| Se | < LOD | 7.97 ± 0.60 | 11.8 ± 2.3 | 7.53 ± 0.95 | < LOD | 4.14 ± 0.26 | 4.90 ± 1.77 | 7.50 ± 1.20 | < LOD | 3.09 ± 1.37 | 6.07 ± 0.27 | 14.7 ± 3.9 |
| Sr | 28.9 ± 5.0 | 64.6 ± 32.2 | 51.0 ± 19.2 | 21.0 ± 6.9 | 26.4 ± 2.3 | 32.0 ± 1.8 | 23.0 ± 3.2 | 24.7 ± 2.4 | 19.9 ± 5.4 | 54.4 ± 9.6 | 27.3 ± 5.8 | 22.0 ± 4.7 |
| V | 0.73 ± 0.15 | 64.3 ± 2.2 | 51.4 ± 5.1 | 61.0 ± 11.6 | 0.81 ± 0.02 | 39.5 ± 4.7 | 24.7 ± 1.8 | 31.4 ± 4.0 | 1.42 ± 0.05 | 53.9 ± 27.3 | 81.9 ± 8.8 | 74.0 ± 4.8 |
| Zn | 25.5 ± 4.8 | 50.8 ± 10.8 | 87.4 ± 26.1 | 32.3 ± 2.2 | 21.3 ± 1.5 | 73.2 ± 1.2 | 78.4 ± 8.8 | 82.4 ± 9.4 | 30.3 ± 0.4 | 29.5 ± 2.3 | 51.7 ± 7.3 | 35.9 ± 2.3 |

< LOD - biomass concentrations were less than the limit of detection (1 mg/kg / 1 ppm for most elements)

All data presented as mean (mg kg^-1^) ± standard error.
