# Supplementary material for: Growth and metal bioconcentration by conspecific freshwater macroalgae cultured in industrial waste water
Source: PeerJ. 2014 May 22;2:e401. doi: 10.7717/peerj.401 (PMC4034596; doi:10.7717/peerj.401)
Supplement: Table S2 [file peerj-02-401-s002.docx]

**Table S2**

|  |  | Barium | | Cobalt | | Chromium | | Copper | | Iron | | Magnesium | |
| --- | --- | --- | --- | --- | --- | --- | --- | --- | --- | --- | --- | --- | --- |
| **Factor** | **DF** | **MS** | **P** | **MS** | **P** | **MS** | **P** | **MS** | **P** | **MS** | **P** | **MS** | **P** |
| Species | 2 | 9137 | 0.60 | 0.47 | 0.08 | 0.003 | 0.80 | 3.92 | 0.35 | 774369 | **0.01** | 349400 | 0.58 |
| Residual | 6 | 1883 |  | 0.12 |  | 0.010 |  | 3.11 |  | 93775 |  | 584065 |  |
|  |  |  |  |  |  |  |  |  |  |  |  |  |  |
|  |  | Manganese | | Lead | | Strontium | | Boron | | Molybdenum | | Vanadium | |
| **Factor** | **DF** | **MS** | **P** | **MS** | **P** | **MS** | **P** | **MS** | **P** | **MS** | **P** | **MS** | **P** |
| Species | 2 | 1439.7 | **0.01** | 0.39 | 0.20 | 270.8 | 0.43 | 2226.9 | 0.35 | 0.21 | 0.59 | 310.4 | 0.20 |
| Residual | 6 | 125.7 |  | 0.19 |  | 274.7 |  | 1755.6 |  | 0.37 |  | 145.1 |  |

Bold P values are statistically significant (P < 0.05).
