# Supplementary figures and images for: Growth and metal bioconcentration by conspecific freshwater macroalgae cultured in industrial waste water
Source: PeerJ. 2014 May 22;2:e401. doi: 10.7717/peerj.401 (PMC4034596; doi:10.7717/peerj.401)

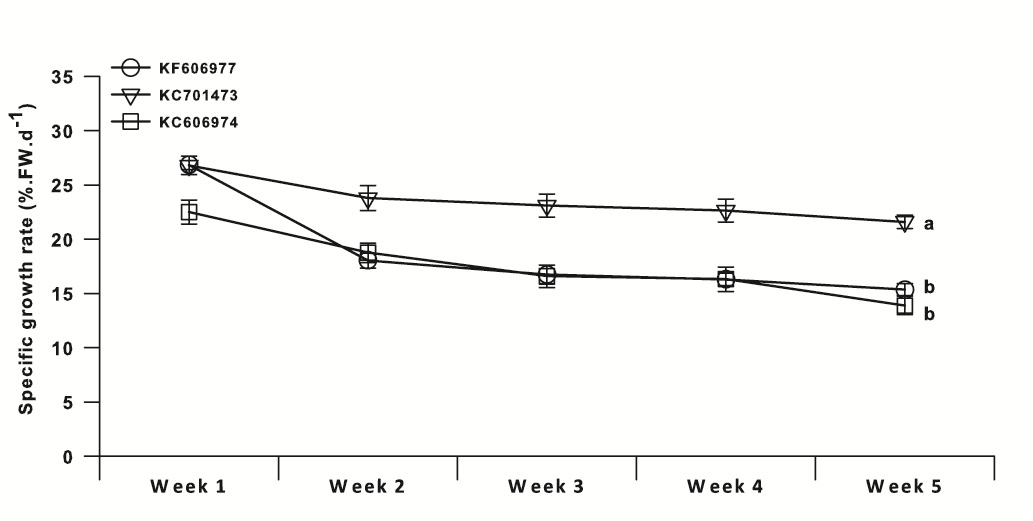

Supplement: Figure S1 [file peerj-02-401-s003.png]

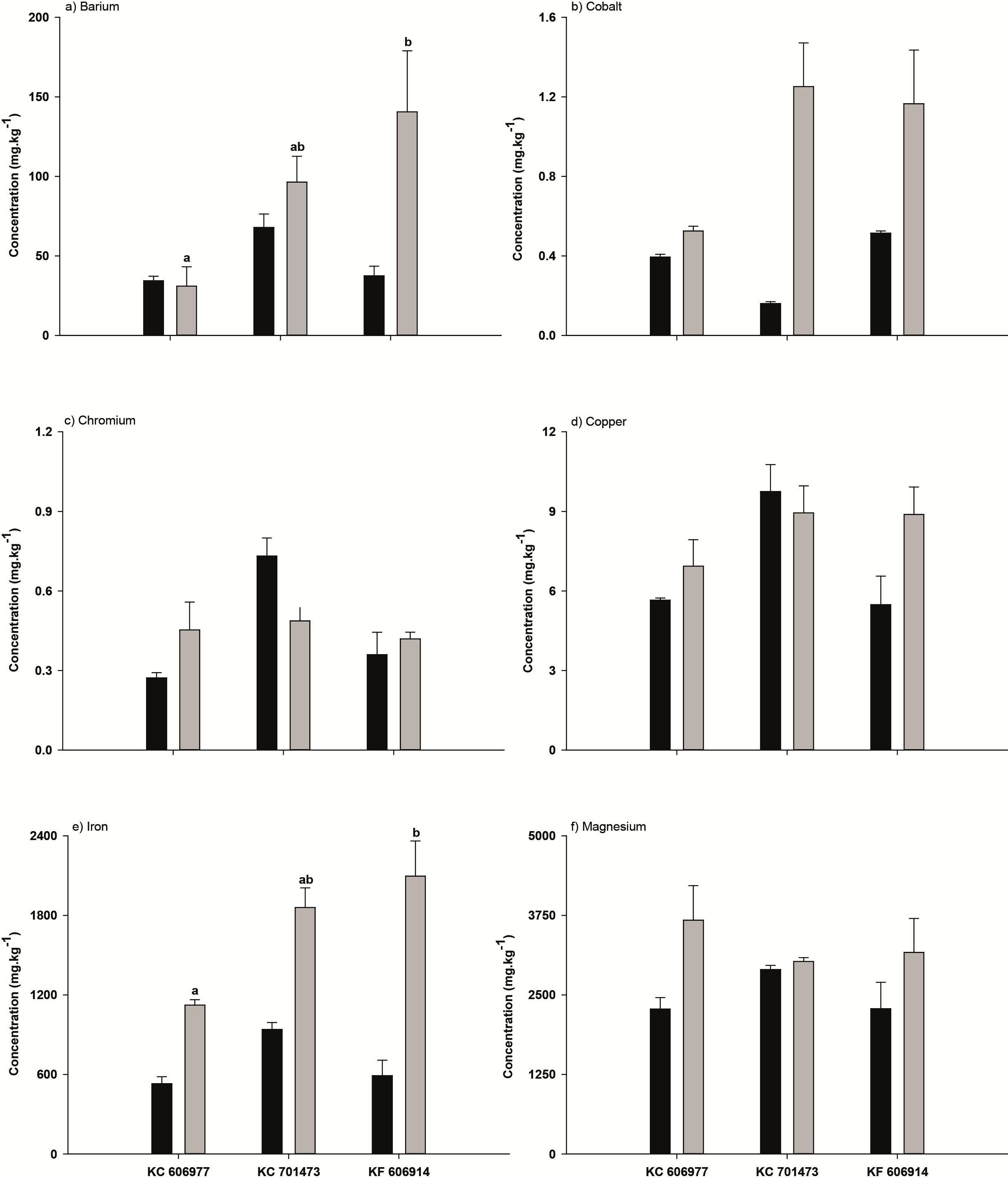

Supplement: Figure S2 [file peerj-02-401-s004.png]

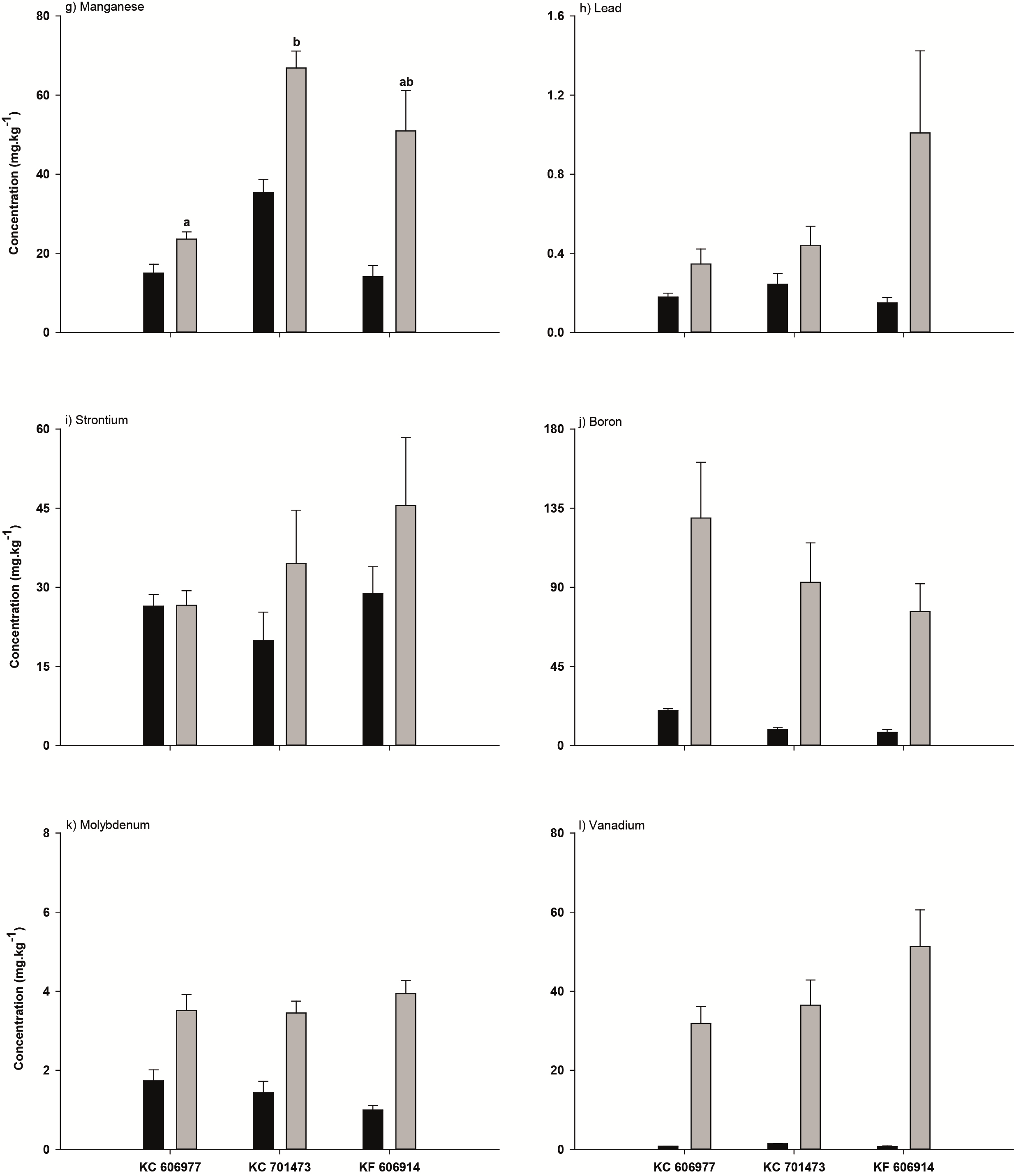

Supplement: Figure S2 continued [file peerj-02-401-s005.png]
